# High rates of multi-drug resistant gram-negative organisms associated with surgical site infections in a teaching hospital in Ghana

**DOI:** 10.1186/s12879-020-05631-1

**Published:** 2020-11-25

**Authors:** Antoinette A. A. Bediako-Bowan, Jørgen A. L. Kurtzhals, Kåre Mølbak, Appiah-Korang Labi, Enid Owusu, Mercy J. Newman

**Affiliations:** 1 Department of Surgery, University of Ghana Medical School, University of Ghana, P. O. Box 4356, Accra, Ghana; 2 Department of Surgery, Korle Bu Teaching Hospital, Accra, Ghana; 3 Department of Veterinary and Animal Science, University of Copenhagen, Copenhagen, Denmark; 4 Division of Infectious Disease Preparedness, Statens Serum Institut, Copenhagen, Denmark; 5 Centre for Medical Parasitology at Department of Immunology and Microbiology, University of Copenhagen, Copenhagen, Denmark; 6 Department of Clinical Microbiology, Copenhagen University Hospital (Rigshospitalet), Copenhagen, Denmark; 7 Department of Microbiology, Korle-Bu Teaching Hospital, P.O. Box 77, Accra, Ghana; 8 Department of Medical Laboratory Sciences, School of Biomedical and Allied Health Science, University of Ghana, Legon, Accra, Ghana; 9 Department of Medical Microbiology, University of Ghana Medical School, University of Ghana, Accra, Ghana

**Keywords:** Multidrug resistant, Surgical site infection, Gram-negative organisms, ESBL, Ghana

## Abstract

**Background:**

There is limited data to guide the prevention and management of surgical site infections (SSI) in low- and middle-income countries. We prospectively studied aetiological agents associated with SSI and their corresponding antibiotic susceptibility patterns in a tertiary hospital in Ghana.

**Methods:**

As part of a cohort study carried out at the surgical department of the Korle Bu Teaching Hospital (KBTH) from July 2017 to April 2019, wound swabs were collected from patients diagnosed with SSI. Isolates cultured from the wound swabs were identified by MALDI TOF and susceptibility testing was conducted according to EUCAST 2020 guidelines. Clinical data were monitored prospectively.

**Results:**

Of 4577 patients, 438 developed an SSI and 352 microbial isolates were cultured. Isolates were predominantly Gram negative (286, 81%), a pattern seen for all kinds of surgery and all wound classes. The most common species included *Escherichia coli*, *Pseudomonas aeruginosa*, *Klebsiella pneumoniae*, *Staphylococcus aureus* and *Acinetobacter baumannii*. The majority of organisms were multi-drug resistant including 86% of *E. coli*, 52% of *A. baumannii* and 86% of *K. pneumoniae;* and 65% (17/26) of the cefotaxime-resistant *K. pneumoniae* were extended spectrum β-lactamase producing. One of 139 *E. coli*, 15 of 49 *P. aeruginosa*, and 6 of 23 *A. baumannii* were meropenem resistant, but no clonal pattern was found. There was a 1% (5/428) prevalence of methicillin-resistant *S. aureus*.

**Conclusions:**

The predominance of Gram-negative organisms and the high level of multi-drug resistance indicate a need to re-evaluate antibiotic prophylaxis and treatment protocols in surgical practice in low- and middle-income countries.

## Background

Surgical site infections (SSI) is a major type of healthcare associated infections (HCAI), forming as much as 33% of all HCAI in sub-Saharan Africa [1, 2]. A major teaching hospital in Ghana also described an SSI incidence risk of 10% with an associated 9 extra days of stay and $1519 excess cost to the institution per patient [3, 4].

The global spread of antibiotic resistance further compounds the problem of SSI. Infections caused by resistant bacteria lead to poor treatment outcomes. Major drivers of antimicrobial resistance in low- and middle-income countries include inappropriate prescription practices and poor infection control measures [5]. Thus, the prevalence of antibiotic use among surgical patients in sub-Saharan Africa is 24–73% [6], and the majority of these antibiotics are prescribed for prophylaxis and usually beyond the recommended 24-h period [7]. Reasons for this prolonged antibiotic use include a fear of infections due to poor infection control [8].

In Ghana, as in most low- and middle-income countries, antimicrobial treatment of wound infections is mainly empirical due to limited laboratory services in most health facilities and the costs to patients of culture and susceptibility testing, when available [9]. The selection of prophylactic antibiotic therapy is based on the operation site and the normal resident flora. Thus, empirical choice of antibiotics relies on knowledge of the susceptibility patterns of common local pathogens. Absence of this data precludes rational use of antibiotics for treatment and prevention of SSI.

In this study, we prospectively identified the aetiological agents of SSI at the surgical department of a tertiary hospital in Ghana, with emphasis on their antibiotic susceptibility patterns, and related to the patient characteristics.

## Methods

### Study site, patient recruitment and collection of samples

Samples were collected as part of a cohort study at the surgical department of the Korle Bu Teaching Hospital from July 2017 to April 2019. The study design and results (for the period July 2017–December 2018) of the surveillance have been reported elsewhere [3]. All patients who underwent surgery in the unit were followed actively for the occurrence of SSI during admission and post-discharge for 30 days, and infection was defined according to Centers of Disease Control and Prevention (CDC) criteria [3, 10]. Wound contamination and SSI type were classified based on the CDC classification [10]. Implant surgery and surgery with wounds that could not be closed immediately were excluded.

Patients on admission had their change of dressing carried out on their bed in the wards. Post-discharge change of dressing was carried out in dressing rooms situated on each ward. Wound swabs were taken from consecutive patients diagnosed with SSI, either on admission or post discharge for microbiology analysis. The project staff had been trained to carefully collect exudate from the infected surgical site using sterile cotton- tipped applicators (Sterilin, U.K), which were transferred to the microbiology laboratory within an hour of sampling. Preliminary culture and susceptibility results were reported immediately to the physicians for management of patients. Isolates were then frozen at − 80 °C and transported to Copenhagen for confirmatory testing and additional analyses.

### Laboratory analyses

Wound swabs were cultured on blood, chocolate and MacConkey agar plates. Isolates were identified using MALDI TOF Biotyper (Bruker Daltonics, Bremen, Germany). Here, susceptibility testing was conducted according to EUCAST 2020 guidelines [11]. The following discs were used; ampicillin (10 μg), amoxicillin-clavulanate (20/10 μg), gentamicin (10 μg), amikacin (30 μg), ciprofloxacin (5 μg), cefuroxime (30 μg), ceftazidime (10 μg), ceftriaxone (30 μg), cefotaxime (10 μg), tazobactam-piperracillin (30/6 μg), meropenem (10 μg), cefoxitin (30 μg), erythromycin (15 μg), clindamycin (2 μg), sulphamethoxazole-trimethoprim (25 μg), penicillin (1 unit), linezolid (10 μg), tetracycline (30 μg), (all from Oxoid Ltd., Basingstoke, United Kingdom (UK)). Isolates were categorized as “resistant” and “susceptible”, including those “susceptible at increased exposure” also classified as “susceptible” [12]. *E. coli* ATCC 25922 and *Staphylococcus aureus* ATCC 25923 were used as controls for Gram negative and positive panels respectively. *Klebsiella pneumoniae* ATCC 700603 was used as quality control strains for ESBL screening.

Enterobacterales resistant to third generation cephalosporins were screened for production of extended spectrum beta lactamases (ESBL) using the double disc diffusion method [13]. Isolates with phenotypic resistance to meropenem were screened depending on the species. *Pseudomonas* spp. were screened for carbapenemases using GeneXpert (Xpert® Carba-R, Cepheid, France), *Acinetobacter* spp. were screened for OXA-23 using a rapid diagnostic test (OXA-23 K-SeT, Coris, Belgium), and *Acinetobacter* spp. and enterobacterales were screened for carbapenemases using NG-CARBA 5® (Hardy Diagnostics, CA, USA). All *Staphylococcus aureus* isolates were screened for methicillin resistance using cefoxitin. Multidrug resistance (MDR) was defined as resistance to ≥1 antibiotic in ≥3 antibiotic groups [14].

#### Data analysis

For each bacterial agent, the percentage frequency of resistant isolates was determined. The resistance patterns of isolates of the same species were screened visually for possible clonal distribution. Distribution of bacterial types by clinical groups were compared by chi-square test and *p* < 0.05 was considered significant. We used Stata /MP version 15.1 (Stata Corp., College Station, Tx, USA) for the analysis.

## Results

### Demographic and patient characteristics

A total of 4577 patients were included in the study. Of these, 438 (9.6%) patients, developed an SSI. The SSI risk was 9.3% (376/4054) in the general surgical department, 26.5% (31/117) in the department of urology and 7.6% (31/406) in paediatric surgery. The median age of the patients who developed an SSI was 45 years (interquartile range 31–60 years) and 239 (54%) patients were female.

From 382 (87%) of the 438 patients, a wound swab was taken for microbiological analysis. No wound swabs were taken in 13% (56/438) of the cases due to; surveillance team missing the opportunity to take a swab at change of wound dressing or at a relaparotomy for an SSI, missing the diagnosis of an SSI until the attention was drawn to the clinical signs and, wound swabs taken but accidentally sent to a private laboratory for analysis.

### Characteristics of surgical wounds

The proportion of patients who developed an SSI was 5.2% (135/2589) for wounds classified as clean, 10.1% (66/655) for a clean contaminated wound, 12.1% (104/859) for contaminated and 27.4% (130/475) for dirty wounds.

We found 352 isolates in 327 (86%) of the 382 swabs. Most were monoculture, but two different species were cultured from each of 25 swabs. Isolates from clean wounds accounted for 29% (103/352) of isolates, clean contaminated wounds for 14% (48), contaminated wounds for 26% (91), and dirty wounds for 31% (110) of isolated microorganisms.

### Aetiology of surgical site infections

Gram-negative microorganisms constituted 81% (286/352) of the isolates. The five most common microorganisms were *Escherichia coli* (139, 39%), *Pseudomonas aeruginosa* (49, 14%), *Klebsiella pneumoniae* (35, 10%), *Staphylococcus aureus* (33, 9%) and *Acinetobacter baumannii* (23, 6%), accounting for approximately 79% of the isolated organisms (Table 1).

**Table 1:** Microbial isolates from infected surgical sites in a teaching hospital in Ghana

Clinical isolates	N	%	Number of isolates with multidrug resistance (%)	Extended spectrum β-lactamase-producing isolates (%)	Meropenem resistant isolates^a^ (%)	Number of isolates with methicillin resistance (%)
*Escherichia coli*	139	39.5	120 (86%)	50 (36%)	1 (1%)	–
*Pseudomonas* spp*.*	49	13.9	17 (35%)	–	15 (31%)	–
*Klebsiella pneumonia*	35	9.9	30 (86%)	17 (48%)	0	–
*Staphylococcus aureus*	33	9.4	8 (24%)	–	–	5 (15%)
*Acinetobacter baumannii*	23	6.5	12 (52%)	–	6 (26%)	–
*Proteus* spp*.*	21	6.0	5 (24%)	0	0	–
*Staphylococcus haemolyticus*	15	4.3	14 (93%)	–	–	0
*Staphylococcus epidermidis*	11	3.1	10 (91%)	–	–	0
*Enterobacter* spp*.*	11	3.1	5 (45%)	2 (18%)	0	–
*Corynebacterium* spp*.*	4	1.1	1 (25%)	–	–	–
*Candida albicans*	2	0.6	ND	–	–	–
*Achromobacter* spp*.*	2	0.6	1 (50%)	–	0	–
*Stenotrophomonas maltophilia*	2	0.6	0	–	0	–
*Providencia stuartii*	2	0.6	0	–	0	–
*Staphylococcus lugdunensis*	1	0.3	0	–	–	0
*Alcaligenes faecalis*	1	0.3	1 (100%)	–	–	–
*Morganella morganii*	1	0.3	1 (100%)	–	0	–

^a^ Four *P. aeruginosa* had the *vim* (Verona integron-encoded metallo-β-lactamase) gene and one *A. baumannii* produced OXA-23

Table 2 describes the pathogens by type of surgery. At least one isolate was found in 187 patients (11%) of the 1646 gastrointestinal and other abdominal surgeries, in 12 (8%) of 140 genitourinary and prostate surgeries, 38 (4%) of 907 breast surgeries, 44 (5%) of 866 hernia and scrotal surgeries, 25 (16%) of 157 limb amputations, 9 (3%) of 307 thyroid surgeries and 12 (2%) of 554 other soft tissue surgeries. The ratio between Gram-negative and Gram-positive organisms differed by type of surgery (Table 3, *p* = 0.002). For gastro-intestinal and genito-urinary surgery, *Staphylococcus* spp. constituted < 16% of the isolates, whereas *Staphylococcus* spp. constituted > 25% of the isolates for hernia, breast, soft tissue and thyroid surgery. Despite this difference, Gram-negative organisms constituted ≥66% of isolates for all types of surgery and as much as 87% for gastro-intestinal surgery.

**Table 2:** Distribution of microbial isolates in relation to type of surgical procedure

Procedure performed	Total *n* = 4577	SSI *n* = 438	*E. coli* *n* = 139	Pseudomonas spp. *n* = 49	Klebsiella spp. *n* = 35	*A. baumannii* *n* = 23	Proteus spp. *n* = 21	Enterobacter spp. *n* = 11	*S. aureus* *n* = 33	*CoNS* *n* = 27	Others *n* = 14	No growth *n* = 55	No samples *n* = 56
Gastrointestinal + other abdominal surgery	1646	253	117	15	21	18	4	4	6	16	5	33	33
Hernia + scrotal surgery	866	54	12	4	3	1	6	2	10	6	3	5	5
Breast surgery	907	49	3	13	3	2	6	0	8	3	2	5	6
Limb amputation	157	33	5	7	5	0	0	2	3	1	3	3	5
Genitourinary tract + prostate surgery	140	20	2	5	1	0	1	1	1	0	1	4	4
Other soft tissue surgery	554	16	0	2	2	1	2	1	3	0	0	2	2
Thyroid surgery	307	13	0	3	0	1	2	1	2	1	0	3	1

*SSI* surgical site infections

CoNS include *S. haemolyticus* and *S. epidermidis* and *S. lugdunensis.* Other microbial isolates include *Corynebacterium* spp*., C. albicans, Achromobacter* spp*., S. maltophilia, P. stuartii, A. faecalis and M. morganii*

**Table 3:** Comparison of distribution of Gram negative: Gram positive for type of procedure performed and type of SSI

Clinical characteristics	Total number of positive cultures *n* = 352	Total number of Gram-negative organisms *n* = 286	Gram-negative organisms %	Total number of Staphylococcus spp.(60) + other Gram-positive organisms (4) *n* = 64	Staphylococcus spp. + Other Gram-positive organisms %	Total number of other microbial isolates (fungi) *n* = 2	Other microbial isolates (fungi) %
Procedure performed							
Gastrointestinal + other abdominal surgery	207	181	87.4	24	11.6	2	1.0
Hernia + scrotal surgery	47	31	65.9	16	34.0	0	0.0
Breast surgery	40	29	72.5	11	27.5	0	0.0
Limb amputation	26	21	80.8	5	19.2	0	0.0
Genitourinary tract + prostate surgery	12	10	83.3	2	16.7	0	0.0
Other soft tissue surgery	10	7	70.0	3	30.0	0	0.0
Thyroid surgery	10	7	70.0	3	30.0	0	0.0
Type of SSI							
Superficial	291	228	78.3	61	21.0	2	0.7
Deep	34	31	85.3	3	8.8	0	0.0
Organ space	27	27	100.0	0	0.0	0	0.0

There was a difference between type of wound and the ratio between Gram-negative and Gram-positive organisms (Table 3), and *S. aureus* was only cultured from superficial SSI (Table 4). Conversely, all isolates from organ-space SSI were Gram negative. A much higher proportion of organ/space infections than other types of infections were not cultured, mostly due to the surveillance team missing the opportunity to take a wound swab during a relaparotomy to drain abscesses.

**Table 4:** Distribution of microbial isolates in relation to type of surgical site infection (SSI)

Type of SSI	Number with surgical site infections	*E. coli* *n* = 139	*Pseudomonas spp.* *n* = 49	*Klebsiella spp.* *n* = 35	*A. baumannii* *n* = 23	*Proteus spp.* *n* = 21	*Enterobacter spp.* *n* = 11	*S. aureus* *n* = 33	CoNS *n* = 27	Others *n* = 14	No growth *n* = 55	No samples *n* = 56
Superficial SSI	366 (%)	105 (29%)	42 (11%)	29 (8%)	18 (5%)	18 (5%)	10 (3%)	33 (9%)	24 (6%)	12 (3%)	45 (12%)	30 (8%)
Deep SSI	49 (%)	17 (34%)	3 (6%)	2 (4%)	4 (8%)	2 (4%)	1 (2%)	0	3 (6%)	2 (4%)	7 (14%)	8 (16%)
Organ-space SSI	48 (%)	17 (35%)	4 (8%)	4 (8%)	1 (2%)	1 (2%)	0	0	0	0	3 (6%)	18 (37%)

CoNS include *S. haemolyticus* and *S. epidermidis* and *S. lugdunensis.* Other microbial isolates include *Corynebacterium* spp*., C. albicans, Achromobacter* spp*., S. maltophilia, P. stuartii, A. faecalis and M. morganii*

### Antimicrobial susceptibility patterns

**Fig. 1:**
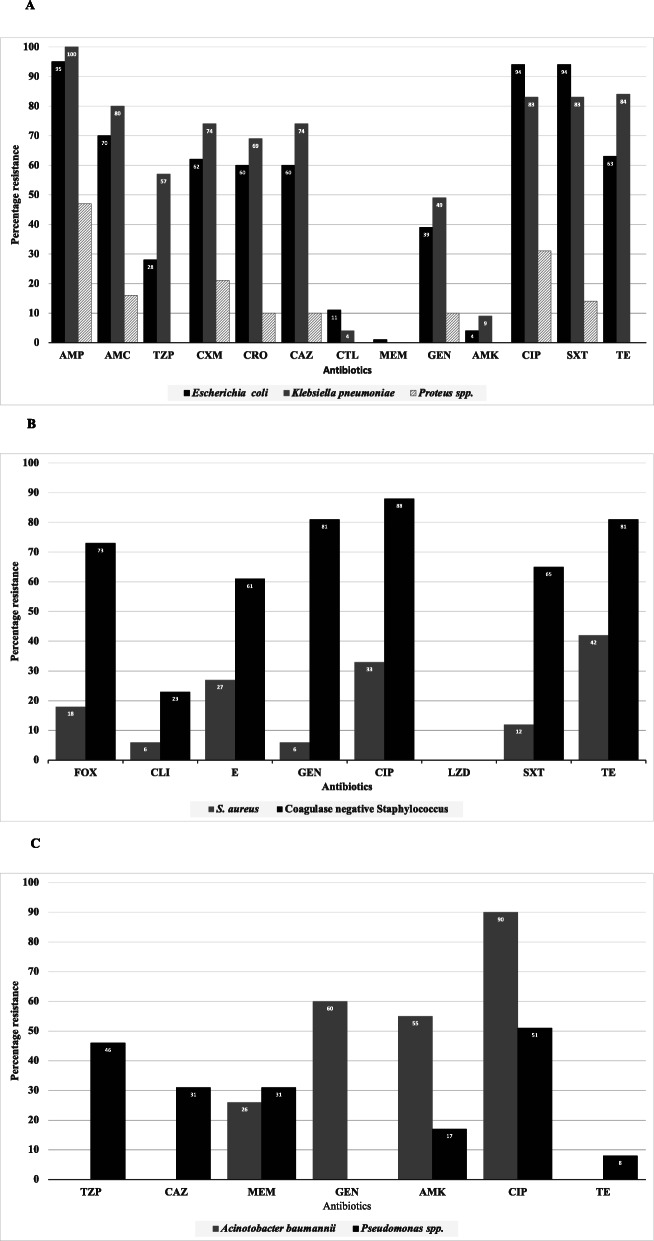
Antibiotic resistance pattern for bacteria isolated from infected surgical sites. **a**, *Eschericia coli, Klebsiella* spp. and *Proteus* spp., **b**, *Pseudomonas aeruginosa* and *Acinetobacter baumannii,* **c**. *Staphylococcus* spp. AMC – amoxicillin/clavulanic acid, AMK – amikacin, AMP – ampicillin, CAZ – ceftazidime, CIP – ciprofloxacin, CLI – clindamycin, CRO – ceftriazone, CTL – cefotaxime + clavulanic acid, CXM – cefuroxime, E – erythromycin, FOX – cefoxitin, GEN – gentamycin, LZD – linezolid, MEM – meropenem, SXT – trimethoprim-sulphamethoxazole, TE – tetracycline, TZP – piperacillin/tazobactam

The majority of bacterial isolates were MDR, ranging from 23 to 86% for *Proteus* spp. and *E. coli* isolates, respectively (Table 1). Acquired resistance to commonly used antibiotics ranged from 1% to meropenem in *E. coli* to 95% to ampicillin, ciprofloxacin and trimethoprim-sulphamethoxazole in *E. coli* (Fig. 1a).

Among *E. coli* and *K. pneumoniae* isolates, 60% (82/139) and 74% (26/35), respectively were resistant to third generation cephalosporins. Sixty-one percent (50/82) and 65% (17/26) of the cefotaxime-resistant *E. coli* and *K. pneumoniae,* respectively were ESBL producing.

Meropenem resistance in this study was mainly found in *Pseudomonas* spp. (15 of 49 isolates) and *Acinetobacter baumannii* (6 of 23 isolates) (Table 1, Fig. 1b). Four *P. aeruginosa* isolates harboured the *vim* gene, encoding Verona integron-encoded metallo-β-lactamase. One *A. baumannii* expressed OXA-23. *K. pneumoniae* showed no resistance to meropenem (Fig. 1a).

Fifteen percent (5/33) of *S. aureus* isolates were methicillin resistant (MRSA). Overall, the MRSA prevalence was 1% (5/438) in the cultured wounds. Resistance of *S. aureus* against other antibiotics ranged from 6% to gentamycin and clindamycin to 42% to tetracycline. Among coagulase-negative staphylococci, the percentage resistance ranged from 23% to clindamycin to 81% to tetracycline. Neither *S. aureus* nor coagulase negative staphylococci showed resistance to linezolid (Fig. 1c).

The sensitivity pattern of the MDR organisms, including those for which specific resistance mechanisms such as ESBL or carbapenemases were detected, did not give any indication of an outbreak of one or more clones (data not shown).

## Discussion

In this study, from a surgical facility in Africa, Gram-negative rods were the primary aetiology of SSI. Despite differences in ratios between Gram-negative and Gram-positive organisms by types of surgery and SSI types, Gram-negatives dominated in all categories. There was a high level of MDR among isolated organisms, including carbapenem-resistant *P. aeruginosa* and *A. baumannii* and ESBL-producing *E. coli* and *K. pneumoniae.* There was no phenotypic indication of clonality of the MDR organisms, indicating that the findings could not be explained by an outbreak. These findings challenge the standard recommendations for empiric prevention and treatment of SSI in low- and middle-income countries.

Unlike earlier studies in Ghana, which recorded low rates of microbiology testing [9], this study recorded a high rate of testing. This can be explained by the fact that under the active study conditions, wound swab samples were taken by the surveillance team when an SSI was diagnosed, irrespective of the clinicians’ diagnosis, and the cost of the testing was also borne by the study. In low- and middle-income countries, low rates of microbiology testing are often reported, presumably due to the limited facilities for microbiology testing, the costs of testing, limited numbers of trained personnel, and the tendency for clinicians to underutilize existing microbiology facilities [9, 15–18] As reported previously, the active surveillance and access to microbiological testing in this study lead to immediate improvements in SSI rates in the course of the study [3].

*E. coli* was the commonest isolated organism, whereas, *S. aureus* and coagulase negative staphylococci constituted less than a fifth of the isolates. Some studies have also predominantly isolated *E. coli* from infected surgical sites post abdominal surgeries [19, 20] but in most studies in Africa, *S. aureus* was the most common pathogen isolated from SSI [21]. The large numbers of gastrointestinal surgeries performed in this study cannot fully explain the predominance of *E. coli* since Gram-negative rods were the predominant aetiology of SSI in all forms of surgery. A possible explanation of this could be a high rate of skin carriage of Gram-negative organisms as shown in a study from Tanzania [22]. In addition, the routine use of antibiotic prophylaxis with an effect on *S. aureus* in our department coupled with a relatively low rate of methicillin resistance may also have skewed the distribution of microorganisms toward Gram negatives. Finally we recently found high levels of antimicrobial air contamination in our surgical facility and demonstrated a causal relationship with SSI [23]. This study indicated that air contamination may have contributed to infections with environmental bacteria such as *P. aeruginosa* and *A. baumannii,* whereas enterobacterales were uncommon in the air samples.

Skin carriage of Gram-negative organisms may be associated with previous antibiotic use. In line with this, it is likely our patients had received antibiotic therapy at referring facilities before coming to our tertiary hospital, where the treatment may have been continued. We have recently documented long periods of administration of antibiotics in surgical units in Ghana, at all levels of health facilities [9]. Long periods of administration of antibiotics have also been reported in other health facilities in low- and middle-income countries [24, 25].

The high usage of antibiotics coupled with the low rate of microbiology testing to inform choice of antibiotic therapy, may explain the high levels of MDR. Hospital-based antimicrobial stewardship programs are said to decrease antibiotic use, though data on this is limited in low- and middle-income countries [26]. There is a need to develop and document the effect of functioning antibiotic stewardship programs based on longitudinal monitoring of microbiological test results from the surgical department.

The choice of antibiotics in the department is essentially based on the Ghana standard treatment guidelines, usually reviewed at intervals of 5 years or more [27, 28].

Antibiotics like ciprofloxacin are used routinely by surgeons as therapeutic and prophylactic treatment for gastrointestinal surgery, and in combination with clindamycin for limb amputation for dry and wet lower limb gangrene. The high prevalence of ciprofloxacin resistance in this study, ranging between 95 and 33% among *E. coli* and *S. aureus* respectively, shows the evolving pattern of resistance. In comparison, a 10% resistance to ciprofloxacin in fecal *E. coli* has been reported in the past [29]. Conversely, the low resistance to vancomycin, piperacillin/tazobactam and linezolid may reflect their low usage or unavailability in most Ghanaian facilities, based on the essential medicines list of the ministry of health [27].

The low prevalence of MRSA confirms previous findings from inpatients in our institution [30]. A high use of amoxicillin/clavulanic acid may explain the low frequency of *S. aureus* in our study, but the high prevalence of amoxicillin/clavulanic acid-resistant Gram-negative organisms suggests the need to re-evaluate the protocols.

The resistance to 3rd generation cephalosporins by *K. pneumoniae* and *E. coli* in our study was mainly caused by ESBL. This cannot be explained by a high usage of third generation cephalosporins as the Ghana treatment guidelines do not recommend this drug class. We have recently reported clonal outbreaks of MDR *K. pneumoniae* in neonatal intensive care units in Ghana [31], but in the present study we did not find indication of ongoing outbreaks, based on phenotypic analysis. The high ESBL rate may thus mimic antibiotic resistance in patients’ own flora indicating widespread carriage of resistant organisms in the society [32].

This study involves data from only one hospital, limiting the generalization of the results, though the hospital serves as a major referral center for a population of over 30 million.

## Conclusion

Gram-negative organisms with a high level of MDR were the predominant organisms isolated from SSI. Antibiotic treatment protocols including prophylactic strategies need to be re-evaluated to improve outcomes and minimize the emergence of antimicrobial resistance.

## Data Availability

Data is available from corresponding author upon request.

## Abbreviations

CDC: Centers for Disease Control and Prevention
Corp.: Corporation
ESBL: Extended spectrum beta lactamases
HCAI: Healthcare associated infections
KBTH: Korle Bu Teaching Hospital
MDR: Multidrug resistant
MRSA: Methicillin resistant *Staphylococcus aureus*
SSI: Surgical site infections
Tx.: Texas
UK: United Kingdom
USA: United State of America
